# The impact of physical activities on positive emotions in children and adolescents: a meta-analysis

**DOI:** 10.3389/fpsyg.2026.1792960

**Published:** 2026-04-24

**Authors:** Zhengyang Zhao, Zhihao Feng, Tong Wang, Jiaxin Deng, Yongfeng Liu

**Affiliations:** School of Sports Training, Chengdu Sport University, Chengdu, Sichuan, China

**Keywords:** children and adolescents, intervention studies, meta-analysis, physical activity, positive emotions

## Abstract

**Background:**

During growth and development, emotional experiences change dynamically. In adolescence, the stability of positive emotions may decline. Exploring whether physical activity can promote positive emotional and related psychological outcomes in children and adolescents has therefore become an important topic in public health and developmental research.

**Objective:**

This review aimed to clarify the effects of physical activity interventions on positive emotional and related psychological outcomes among children and adolescents aged 7–25 years through a meta-analysis.

**Methods:**

Relevant studies were systematically retrieved from PubMed, Web of Science, EBSCO, Embase, Cochrane Library, and PsycINFO up to December 12, 2025. The search strategy combined terms related to physical activity, positive emotional and related psychological outcomes, children and adolescents, and randomized controlled trials. Because the included studies used different outcome measures and scoring ranges, standardized mean differences (SMDs) with 95% confidence intervals were calculated using a random-effects model. Subgroup analyses were conducted according to outcome type, intervention duration, intervention type, control-group type, session length, and age group.

**Results:**

15 studies comprising 16 comparisons were included in the meta-analysis. The pooled analysis showed that physical activity interventions were associated with a significant improvement in positive emotional and related psychological outcomes among children and adolescents (SMD = 0.72, 95% CI 0.33 to 1.12, *P* = 0.0003). However, substantial heterogeneity was observed across studies (*I*^2^ = 95%). Subgroup analyses showed significant pooled effects for self-esteem, psychological wellbeing, aerobic exercise, no-treatment controls, interventions shorter than 12 weeks, and sessions lasting 30–60 min, whereas positive mood, mind-body exercise (yoga, Tai Chi, and Baduanjin), mixed exercise, and treatment-as-usual controls did not show significant pooled effects.

**Conclusion:**

The available evidence suggests that physical activity interventions may improve positive emotional and related psychological outcomes in children and adolescents. However, given the substantial heterogeneity and conceptual differences among outcome measures, these findings should be interpreted cautiously. The subgroup findings were derived from separate analyses and should not be interpreted as defining a single optimal intervention package.

**Systematic Review Registration:**

identifier: CRD420261279752.

## 1 Introduction

The global prevalence of mental health issues among children and adolescents currently represents a significant public health challenge. According to the World Health Organization, emotional and behavioral disorders constitute the leading cause of the disease burden among adolescents aged 10–19 years (World Health Organization, 2021, *Mental health of adolescents*). This crisis has been further exacerbated in the post-pandemic era by factors such as social isolation and academic pressure (Racine et al., 2021). Against this backdrop, positive emotions are not merely a unidimensional indicator of psychological wellbeing but a core element of individual flourishing. Drawing on Fredrickson's Broaden and Build Theory, positive emotions expand an individual's cognitive-behavioral repertoire and foster long-term psychological resources, thereby enhancing social adaptability and resilience and serving as a key protective factor against depression and anxiety (Fredrickson, 2013). Consequently, identifying modifiable factors that effectively cultivate positive emotions is critical for developing adolescent mental health promotion strategies.

Physical activity has been widely recognized as a promising intervention with multidimensional benefits for physical and mental health. Substantial evidence suggests that exercise not only improves cardiorespiratory function and metabolic health but is also associated with emotional and psychological outcomes through a range of physiological, psychological, and social pathways (Biddle et al., 2019; Lubans et al., 2016). At the physiological level, physical activity stimulates the central nervous system to release endocannabinoids, endorphins, and monoamine neurotransmitters (dopamine, serotonin), thereby inducing immediate pleasurable and anxiolytic effects (Basso and Suzuki, 2017). Moreover, exercise-induced increases in brain-derived neurotrophic factor (BDNF) contribute to hippocampal neuroplasticity, which plays a vital role in mood regulation and learning capacity (Mandolesi et al., 2018). From a psychosocial perspective, participation in group-based or school-related sports provides adolescents with essential social connections, peer support, and a sense of achievement and self-efficacy—all of which are key sources of positive emotions (Eime et al., 2013; Lubans et al., 2016). In this review, the term “physical activity intervention” was used as an umbrella term to include structured exercise, school-based activity programs, and selected sport-related movement interventions where these met the eligibility criteria.

Among adult populations, the positive effects of physical exercise on emotional wellbeing have been substantiated by multiple high-quality meta-analyses (Rebar et al., 2015; Schuch et al., 2016). However, when examining children and adolescents, a demographic undergoing rapid developmental changes, the evidence becomes more complex and less conclusive. Although systematic reviews have reported associations between physical activity and lower levels of depressive and anxiety symptoms, as well as higher life satisfaction and happiness in youth (Biddle et al., 2019; Rodriguez-Ayllon et al., 2019), there remains a scarcity of synthesized research focusing specifically on positive emotions as a core construct within positive psychology, such as joy, self-esteem, and wellbeing. Existing studies yield heterogeneous outcomes, which may be moderated by a range of factors, including developmental stage (reflecting neurodevelopmental and psychosocial differences between childhood and adolescence) (Pfeifer and Blakemore, 2012), type of exercise (varying psychosocial benefits of aerobic vs. team-based activities) (Lubans et al., 2016), dose-response relationships (frequency, intensity, duration) (Janssen and Leblanc, 2010), and individual or contextual factors (gender, baseline mental health status, familial and socioeconomic support) (Vella et al., 2015).

Notably, several critical gaps persist in the current literature. First, the majority of reviews and meta-analyses focus predominantly on the reduction of negative psychological symptoms, with limited synthesis dedicated to enhancements in positive emotional states and psychological functioning (Biddle and Asare, 2011; Cairney et al., 2019; Rodriguez-Ayllon et al., 2019). Second, the underlying mechanisms, such as the interplay of neurobiological, psychosocial, and behavioral pathways, have not been systematically elucidated or empirically integrated within child and adolescent populations (Lubans et al., 2016). Third, many earlier reviews are methodologically limited, often relying on cross-sectional designs and failing to incorporate recent high-quality randomized controlled trials and longitudinal cohort studies (Singh et al., 2019). Furthermore, the moderating role of cultural context is frequently overlooked, despite potential variations in the emotional impact of physical activity across different sociocultural environments.

From a theoretical perspective, the relationship between physical activity and positive emotions in youth may be understood through several complementary frameworks. Fredrickson's broaden-and-build theory (Fredrickson, 2004) suggests that positive emotions broaden cognitive and behavioral repertoires and help build enduring personal resources. In parallel, social cognitive theory highlights the role of mastery experiences and self-efficacy in shaping emotional responses to behavior. More specifically, the conceptual model proposed by Lubans et al. (2016) indicates that physical activity may influence mental health through neurobiological, psychosocial, and behavioral pathways. These frameworks together provide a rationale for examining not only whether physical activity improves positive emotions, but also under what conditions such effects may vary across intervention types and developmental stages.

Specific objectives included: first, quantitatively synthesizing the overall effect size of physical activity interventions on positive emotional and related psychological outcomes in children and adolescents; second, performing subgroup analyses to examine potential moderators such as age, intervention type, intervention duration, and session length; and finally, discussing possible factors that may influence intervention effects. In this review, positive outcomes were operationalized broadly to include positive affective states and closely related psychological constructs, such as positive mood, psychological wellbeing, self-esteem, and self-efficacy.

## 2 Materials and methods

Following the guidelines outlined in the Preferred Reporting Items for Systematic Reviews and Meta-Analysis (PRISMA) and the Cochrane Handbook for Systematic Reviews and Meta-Analysis (Moher et al., 2010), this review was conducted. Moreover, the protocol for this review was duly registered on PROSPERO under the registration number CRD420261279752.

### 2.1 Search strategy

The review was conducted in accordance with PRISMA guidance and relevant Cochrane recommendations (Cumpston et al., 2019; Moher et al., 2010). Systematic searches were conducted in PubMed, Web of Science, EBSCO, Embase, the Cochrane Library, and PsycINFO from database inception to December 12, 2025. The search strategy combined terms from four domains: (1) physical activity or exercise, (2) positive emotional and related psychological outcomes, (3) children or adolescents, and (4) randomized controlled trials. Terms within each domain were combined with OR, and the four domains were combined with AND. The search strategy was a Boolean logic search with the following search strategies: (“physical activity” or “physical exercise” or “physical activity” or “sport movement” or “sport” or “motor” or “aerobic exercise” or “aerobic training” or “resistance exercise” or “strength training” or “muscle-strengthening exercise” or “physical education” or “fitness game”) AND (“affect” or “mood” or “emotion” or “happiness” or “pleasure” or “enjoyment” or “subjective wellbeing” or “self-esteem”) AND (“randomized controlled trial” or “RCT”) AND (“adolescent” or “teen” or “teenager” or “student” or “juvenile” or “school-aged children”). Positive outcomes were operationalized using terms related to positive affect, mood, enjoyment, happiness, self-esteem, self-efficacy, and psychological wellbeing. The detailed search strategy was presented in Table 1, demonstrating the meticulousness and accuracy of our research approach. The searches were conducted independently by two researchers (ZYZ and ZHF), with a third researcher (YFL) consulted in case of disagreement. Study screening and data extraction were conducted by two reviewers, and disagreements were resolved through discussion with a third reviewer where necessary.

**Table 1:** Summary of search terms.

Category		Included search terms
Physical activity	OR	“physical exercise” or “sports activities” or “sport movement” or “sport” or “motor” or “athletic sports” or “aerobic exercise” or “aerobic training” or “resistance exercise” or “strength training” or “muscle-strengthening exercise” or “physical education” or “fitness game”
AND		
Positive emotion	OR	“Affect” or “mood” or “emotion” or “happiness” or “pleasure” or “enjoyment” or “subjective well-being” or “self-esteem”
AND		
Adolescent	OR	“teen” or “teenager” or “student” or “juvenile” or “school-aged children”
AND		
Randomized controlled trial	OR	“randomized controlled trial” or “RCT”

### 2.2 Eligibility criteria

The relevant studies' inclusion criteria were established following the PICOS framework. For participants (*P*), eligible studies included children, adolescents, and young adults aged under 25 years. Regarding the intervention measures (I), the experimental group received various forms of physical activity interventions, including aerobic exercises, resistance training, and fitness games. The control-group (*C*) included no treatment (NT), treatment as usual (TAU), or attention/activity placebo (AP). The main outcome (*O*) focused on positive emotional and related psychological outcomes in children and adolescents. Study design (*S*) was limited to randomized controlled trials. Exclusion criteria included (1) non-English literature, as well as unpublished materials, dissertations, conference abstracts, and review articles; (2) studies with research subjects being adults, animals, or special populations (such as those with neurodevelopmental disorders, physical disabilities, or mental illnesses); (3) literature lacking valid data extraction; (4) duplicate publications; and (5) full texts that were inaccessible.

### 2.3 Data extraction

Data extraction and study selection were conducted in accordance with the PRISMA statement. EndNote 20 was used to import records from all databases and remove duplicates. Study screening and data extraction were performed independently by two reviewers (ZYZ and ZHF), and disagreements were resolved through discussion with a third reviewer (YFL). The extracted data was analyzed by ZYZ, ZHF, and JXD, under the supervision of TW and YFL. When relevant data could not be extracted, we contacted the corresponding authors for clarification. If no response was obtained, the study was excluded from quantitative synthesis. Although a formal inter-rater coefficient was not calculated, consensus procedures were applied throughout the review process to minimize selection bias. The extracted data included the first author, publication year, participant characteristics (sample size, age, country, and region), sampling type, study design, intervention measures, assessment tools, and outcome indicators (Table 2).

**Table 2:** Study characteristics.

Author (Year)	Country	Age (mean)	Sample characteristics (exp/ctrl)	Intervention measure	Intervention duration (weeks)	Session length (min)	Outcome Measurement Tools	Outcome	Control group
Ahmed et al. (2023)	Australia	14.3	160/160	Aerobic	12	30	Cantril Ladder	Psychological well-being	NT
Andrade et al. (2020)	Brazil	9.41	68/72	Aerobic	8	40	BRUMS	Positive mood	TAU
Asadabadi and Karami (2025)	Iran	7–10	52/52	Aerobic	8	30	CSEI	Self-esteem	NT
Bonhauser et al. (2005)	Chile	15.54	98/100	Aerobic	30	90	TSCS	Self-esteem	TAU
Brand et al. (2019)	Switzerland	13	35/34	Aerobic	NR	35	A computerized test	Positive mood	AP
Castellote-Caballero et al. (2024)	Spain	20.29	65/64	Mind-body exercise	12	60	WEMWBS	Psychological well-being	NT
Costigan et al. (2016)	Australia	14–16	21/22/22	Mixed	8	8–10	FS	Psychological well-being	TAU
Halliwell et al. (2018)	England	9–11	190/154	Mind-body exercise	4	40	PANAS-C	Positive mood	TAU
Kavosi et al. (2015)	Iran	18–22	45/45	Aerobic	8	45	CSEI	Self-esteem	NT
Li et al. (2015)	China	18–25	101/105	Mind-body exercise	12	60	GSES	Self-efficacy	TAU
Li and Zhou (2025)	China	14–18	30/30	Mixed	8	15	MSPYA	Self-esteem	AP
Noggle et al. (2012)	United States of America	17.1	36/15	Mind-body exercise	10	30–40	PANAS-C	Positive mood	TAU
da Silva et al. (2025)	Brazil	13.6	165/141	Mixed	12	20	KIDSCREEN-27	Psychological well-being	TAU
Telles et al. (2013)	India	10.4	49/49	Mind-body exercise	12	45	BSEQ	Self-esteem	TAU
Zheng et al. (2015)	China	16–25	95/103	Mind-body exercise	12	60	SSES	Self-esteem	NT

Exp, Experiment group; Ctrl, Control group; Cantril Ladder, Cantril Self-Anchoring Striving Scale; BRUMS, Brunel Mood Scale; CSEI, Coopersmith Self-Esteem Inventory; TSCS, Tennessee Self-Concept Scale; FS, Flourishing Scale; WEMWBS, Warwick-Edinburgh Mental Wellbeing Scale; PANAS-C, the Positive and Negative Affect Scale for Children; GSES, the General Self-Efficacy Scale; MSPYA, Multidimensional Scale of Psychological States in Adolescents; KIDSCREEN-27, KIDSCREEN-27 Health-Related Quality of Life Questionnaire; BSEQ, Battle's self-esteem questionnaire; SSES, Schoolagers' Stress and Emotion Scale; NT, No-Treatment; TAU, Treatment as Usual; NR, No report; AP, Attention/Activity Placebo; K, Number of trials; SMD, Standardized Mean Difference; CI, Confidence Interval; PA, Physical Activity.

After data extraction, Review Manager 5.3 (Collaboration, 2014) was used for the meta-analysis. For each included comparison, the post-intervention mean, standard deviation (SD), and sample size of the intervention and control groups were entered whenever these data were available in a comparable form. Where studies also reported baseline values, these were reviewed descriptively to assess pre-intervention comparability. Because the included studies assessed outcomes using different instruments and scoring ranges, standardized mean differences (SMDs) with 95% confidence intervals were calculated as the pooled effect size. A random-effects model was used to account for between-study heterogeneity. Statistical heterogeneity was assessed using the *I*^2^ statistic (Hahn et al., 2000; Cohen, 2013). When studies reported multi-arm designs, the shared control-group was split evenly across comparisons to avoid double counting. When sex-specific outcomes were available, overall group data were preferentially used for the primary analysis, while sex-specific data were reserved for subgroup interpretation where appropriate.

When significant heterogeneity was detected (*I*^2^ > 50%), subgroup analyses were conducted to explore potential sources of between-study variability (Moher et al., 2015), including outcome type, intervention duration, intervention type, control-group type, session length, and age group. Sensitivity analyses were conducted to assess the robustness of the pooled estimates.

### 2.4 Methodological quality assessment

ZYZ and ZHF independently assessed the methodological quality of the included studies using the Cochrane Risk of Bias tool (Cumpston et al., 2019). During this process, disagreements were resolved through discussion. If disagreements persisted, a third reviewer made the final decision. The methodological quality of all included studies was evaluated using the Cochrane Risk of Bias tool, which covers seven items: Random sequence generation (selection bias), allocation concealment (selection bias), subject and personnel blinding (performance bias), outcome evaluation blinding (detection bias), incomplete outcome data (depletion bias), selective reporting (reporting bias), and other biases. Based on the signaling questions, each domain was judged as “low risk,” “unclear risk,” or “high risk” of bias, and an overall judgment was then made for each study.

## 3 Results

### 3.1 Literature screening process and results

**Figure 1:**
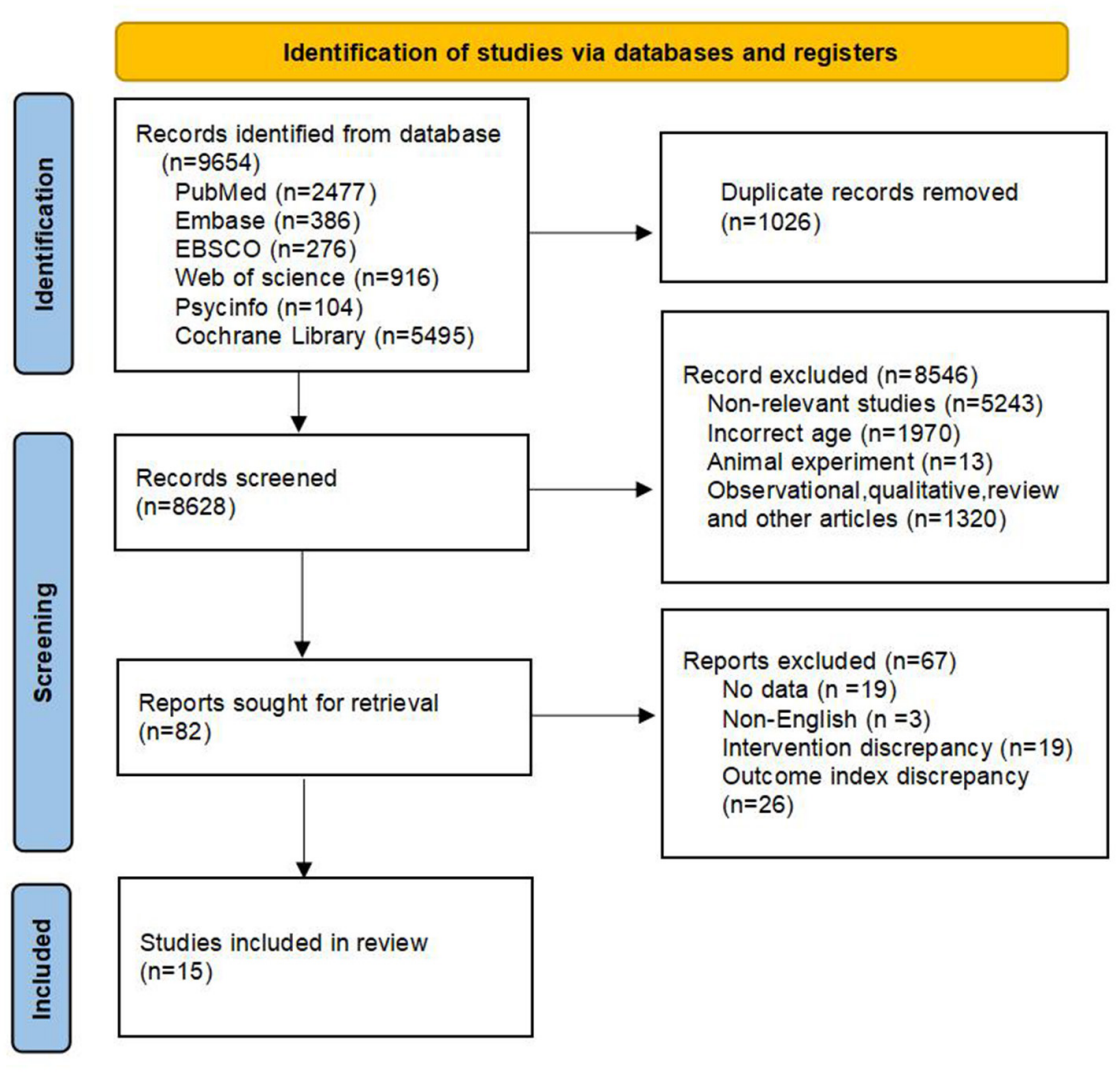
Flow chart of literature retrieval.

After searching for the key terms, 9,654 articles were retrieved from the electronic databases. The literature search results and the research screening process are shown in Figure 1. Initially, 9,654 articles were retrieved from 6 databases, among which 5,495 were from Cochrane Library, 2,488 from PubMed, 916 from Web of Science, 386 from Embase, 276 from EBSCO, and 104 from PsycInfo. The retrieved articles were imported into the literature management software EndNote 21, and duplicate items were deleted, resulting in 8,628 articles. 8,508 articles that did not meet the eligibility criteria during the title and abstract reading and screening stage were excluded. These non-standard papers included irrelevant studies (*n* = 5,243); incorrect age (*n* = 1,970); observational, qualitative, review, and other types of articles (*n* = 1,320); and animal experiments (*n* = 13). Subsequently, the full texts of the remaining 82 articles were read, and non-relevant literature was excluded: outcome indicators did not match (*n* = 26); intervention types did not match (*n* = 19); no data (*n* = 19); not in English (*n* = 3). A total of 15 full texts were obtained, and effect values and 95% confidence intervals or calculable relevant data were obtained in the articles. These were included in the meta-analysis of intervention studies.

### 3.2 Study characteristics

The basic characteristics included in the article are shown in Table 2. The included studies were published between 2005 and 2025. No publication-year restriction was imposed in the search strategy; rather, 2005 was the earliest publication year among the eligible studies. The sample size of the experiments ranged from 51 to 344 participants, and the average age of the subjects ranged from 7 to 25 years old. Among them, 4 studies were for college students, 6 for junior high school and high school students, 4 for primary school students, and 1 study covered the span from primary school to junior high school. This meta-analysis included a total of 15 studies. In terms of sample size distribution, 11 studies (accounting for 73%) had a sample size of more than 100 people, while the remaining 4 studies (accounting for 27%) had a sample size of less than 100 people. In Tables 2, 3, the physical intervention measures implemented in each experiment are summarized, namely aerobic exercise, mind-body exercise (yoga, Tai Chi and Baduanjin), and mixed. Most of them adopted aerobic exercise. The duration of the intervention was 4 to 30 weeks, and the duration of each intervention was 8 to 90 min. The control-group included NT (*n* = 5), TAU (*n* = 8), and AP (*n* = 2).

**Table 3:** Subgroup analyses based on the primary meta-analysis.

Subgroup analysis	*K*	SMD	95%Cl	*p* value	Heterogeneity	Test for subgroup difference
15.5-7.4,-13.5175.3mmPrimary meta-analysis	15	0.72	0.33 to 1.12	*p* < 0.01	X^2^ = 299.67, df = 15(*p* < 0.00001), Z = 3.60, I^2^ = 95%	
Type of exercise intervention (3 sub-group analysis)						
Aerobic	6	1.88	0.81 to 2.94	*p* < 0.01	X^2^ = 232.78, df = 5 (*p* < 0.00001), Z = 3.46, I^2^ = 98%	X^2^ = 12.36, df = 2(*p* = 0.002), Z = 3.60, I^2^ = 83.8%
Mind-body exercise	6	0.03	−0.16 to 0.22	*P* = 0.75	X^2^ = 10.67, df = 5(*p* = 0.06), Z = 0.32, I^2^ = 53%	
Mixed	3	0.29	−0.02 to 0.61	*p* < 0.01	X^2^ = 4.86, df = 3(*p* = 0.18), Z = 1.81, I^2^ = 38%	
Age (2 sub-group analysis)						
≤ 12 years	4	1.57	0.22 to 2.92	*P* = 0.02	X^2^ = 159.61, df = 3(*p* < 0.00001), Z = 2.28, I^2^ = 98%	X^2^ = 2.28, df = 1(*p* = 0.13), Z = 3.60, I^2^ = 56.1%
13–25 years	11	0.49	0.12 to 0.86	*P* = 0.01	X^2^ = 139.95, df = 11(*p* < 0.00001), Z = 2.57, I^2^ = 92%	
Type of control group (3 sub-group analysis)						
PA-NT	5	2.18	0.90 to 3.47	*p* < 0.01	X^2^ = 239.08, df = 4(*p* < 0.00001), Z = 3.33, I^2^ = 98%	X^2^ = 9.65, df = 2 (*p* = 0.008), Z = 3.60, I^2^ = 79.3%
PA-TAU	8	0.13	−0.07 to 0.34	*P* = 0.19	X^2^ = 23.82, df = 8 (*p* = 0.002), Z = 1.31, I^2^ = 66%	
PA-AP	2	0.34	−0.50 to 1.19	*p* = 0.43	X^2^ = 5.74, df = 1(*p* = 0.02), Z = 0.80, I^2^ = 83%	
Duration (2 sub-group analysis)						
≥12 weeks	7	0.2	−0.02 to 0.43	*P* = 0.07	X^2^ = 26.46, df = 6(*p* = 0.0002), Z = 1.78, I^2^ = 77%	X^2^ = 5.20, df = 1 (*p* = 0.02), Z = 3.71, I^2^ = 80.8%
<12 weeks	7	1.45	0.40 to 2.49	*p* < 0.01	X^2^ = 260.40, df = 7 (*p* < 0.00001), Z = 2.71, I^2^ = 97%	
Time (3 sub-group analysis)						
<30 min	3	0.29	−0.02 to 0.61	*P* = 0.07	X^2^ = 4.86, df = 3 (*p* = 0.18), Z = 1.81, I^2^ = 38%	X^2^ = 3.97, df = 2 (*p* = 0.14), Z = 3.60, I^2^ = 49.6%
30–60 min	11	0.93	0.37 to 1.49	*p* < 0.01	X^2^ = 291.75, df = 10 (*p* < 0.00001), Z = 3.25, I^2^ = 97%	
>60 min	1	5.24	2.50 to 7.98	*p* < 0.01	-	
Outcome (4 sub-group analysis)						
Self-efficacy	1	−0.31	−0.59 to −0.04	*P* = 0.03	–	X^2^ = 22.98, df = 3 (*p* < 0.0001), Z = 3.65, I^2^ = 86.9%
Positive mood	4	0.13	−0.15 to 0.41	*P* = 0.37	X^2^ = 6.71, df = 3 (*p* = 0.08), Z = 0.89, I^2^ = 55%	
Psychological well-being	4	0.31	0.16 to 0.47	*p* < 0.01	X^2^ = 4.53, df = 4(*p* = 0.34), Z = 3.94, I^2^ = 12%	
Self-esteem	6	2.31	0.81 to 3.80	*p* < 0.01	X^2^ = 232.82, df = 4(*p* < 0.00001), Z = 3.02, I^2^ = 98%	

### 3.3 Risk of bias

**Figure 2:**
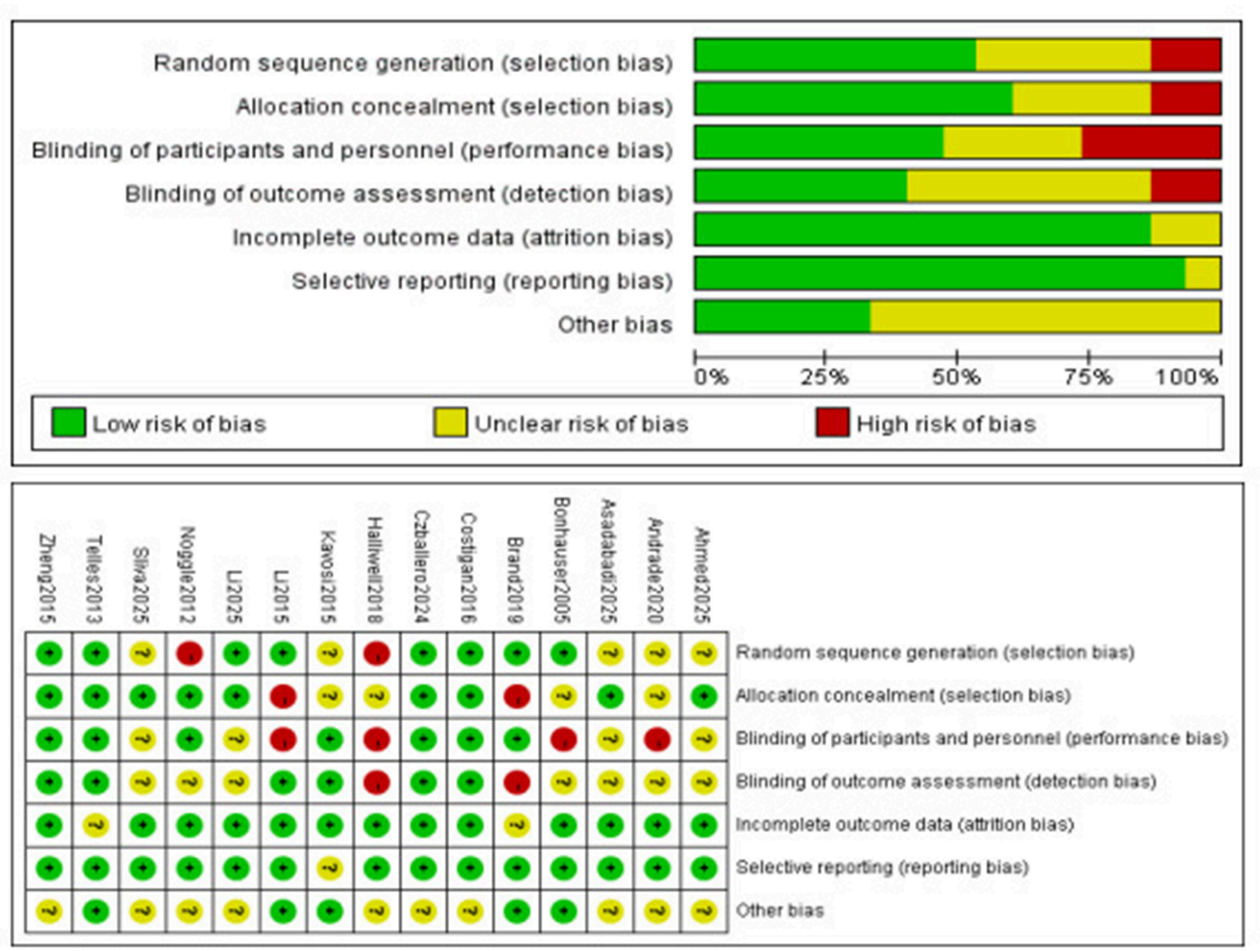
Results of the Cochrane risk of bias tool.

The methodological quality of the included studies was assessed using the Cochrane Risk-of-Bias tool, which comprises seven evaluation criteria. Regarding random sequence generation, 8 studies (Bonhauser et al., 2005; Brand et al., 2019; Castellote-Caballero et al., 2024; Costigan et al., 2016; Li et al., 2015; Li and Zhou, 2025; Telles et al., 2013; Zheng et al., 2015) were rated as low risk, 5 (Ahmed et al., 2023; Andrade et al., 2020; Asadabadi and Karami, 2025; da Silva et al., 2025; Kavosi et al., 2015) as unclear risk, and 2 (Halliwell et al., 2018; Noggle et al., 2012) as high risk. In terms of allocation concealment, 9 (Ahmed et al., 2023; Asadabadi and Karami, 2025; Castellote-Caballero et al., 2024; Costigan et al., 2016; da Silva et al., 2025; Li and Zhou, 2025; Noggle et al., 2012; Telles et al., 2013; Zheng et al., 2015) studies were classified as low risk, 4 (Andrade et al., 2020; Bonhauser et al., 2005; Halliwell et al., 2018; Kavosi et al., 2015) as unclear risk, and 2 (Brand et al., 2019; Li et al., 2015) as high risk. For performance bias, 7 (Brand et al., 2019; Castellote-Caballero et al., 2024; Costigan et al., 2016; Kavosi et al., 2015; Noggle et al., 2012; Telles et al., 2013; Zheng et al., 2015) studies were deemed low risk, 4 (Ahmed et al., 2023; Asadabadi and Karami, 2025; da Silva et al., 2025; Li and Zhou, 2025) as unclear risk, and 4 (Andrade et al., 2020; Bonhauser et al., 2005; Halliwell et al., 2018; Li et al., 2015) as high risk. Concerning detection bias, 6 (Castellote-Caballero et al., 2024; Costigan et al., 2016; Kavosi et al., 2015; Li et al., 2015; Telles et al., 2013; Zheng et al., 2015) studies were rated as low risk, 7 (Ahmed et al., 2023; Andrade et al., 2020; Asadabadi and Karami, 2025; Bonhauser et al., 2005; da Silva et al., 2025; Li and Zhou, 2025; Noggle et al., 2012) as unclear risk, and 2 (Brand et al., 2019; Halliwell et al., 2018) as high risk. With respect to attrition bias, 13 studies (Ahmed et al., 2023; Andrade et al., 2020; Asadabadi and Karami, 2025; Bonhauser et al., 2005; Castellote-Caballero et al., 2024; Costigan et al., 2016; da Silva et al., 2025; Halliwell et al., 2018; Kavosi et al., 2015; Li et al., 2015; Li and Zhou, 2025; Noggle et al., 2012; Zheng et al., 2015) were evaluated as low risk and 2 (Brand et al., 2019; Telles et al., 2013) as unclear risk. In the domain of reporting bias, 14 studies (Ahmed et al., 2023; Andrade et al., 2020; Asadabadi and Karami, 2025; Bonhauser et al., 2005; Brand et al., 2019; Castellote-Caballero et al., 2024; Costigan et al., 2016; da Silva et al., 2025; Halliwell et al., 2018; Li et al., 2015; Li and Zhou, 2025; Noggle et al., 2012; Telles et al., 2013; Zheng et al., 2015) were categorized as low risk and 1 (Kavosi et al., 2015) as unclear risk. Finally, for other potential biases, 5 studies (Bonhauser et al., 2005; Brand et al., 2019; Kavosi et al., 2015; Li et al., 2015; Telles et al., 2013) were considered low risk and 10 (Ahmed et al., 2023; Andrade et al., 2020; Asadabadi and Karami, 2025; Castellote-Caballero et al., 2024; Costigan et al., 2016; da Silva et al., 2025; Halliwell et al., 2018; Li and Zhou, 2025; Noggle et al., 2012; Zheng et al., 2015) as unclear risk (Figure 2).

**Figure 3:**
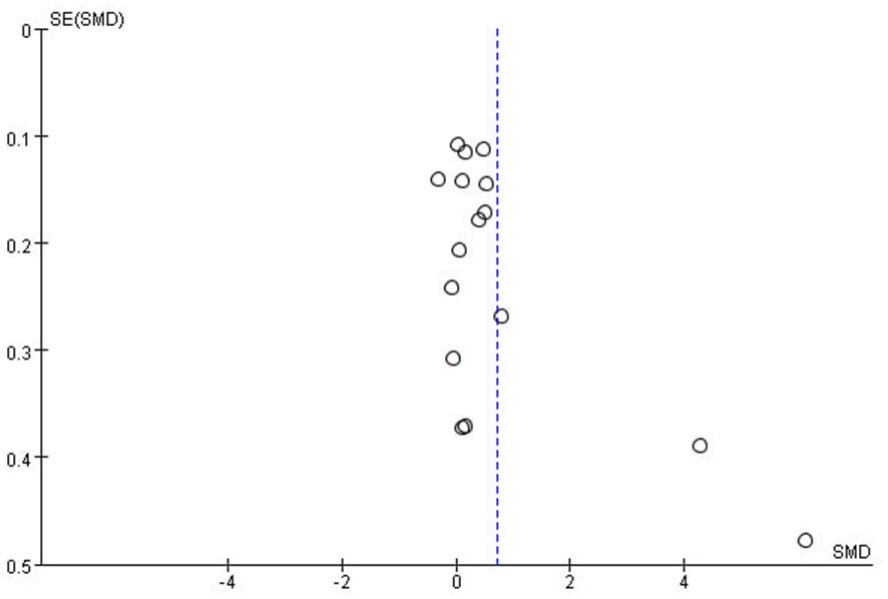
Funnel plot publication bias in the relationship between PA and positive emotions in children and adolescents.

Publication bias was assessed for the included studies. As illustrated in Figure 3, the funnel plot exhibited asymmetry, suggesting the possible presence of publication bias. However, this interpretation relies predominantly on subjective judgment and may lack precision. Evaluation of publication bias was visually performed via funnel plot analysis across the included studies. The funnel plots demonstrated noticeable asymmetry, indicative of potential publication bias. Such asymmetry implies that studies with non-significant results may be underrepresented, potentially leading to an overestimation of the true effect size in our meta-analysis. Aside from publication bias, heterogeneity in study designs—including variations in intervention frequency, intensity of exercise interventions, and demographic characteristics of the participant populations—may also contribute to the observed asymmetry in the funnel plots. Moreover, smaller studies often exhibit more variable effect sizes, further exacerbating the asymmetry.

### 3.4 Meta-analysis result

#### 3.4.1 The relationship between participation of children and adolescents in physical activity and positive emotions

**Figure 4:**
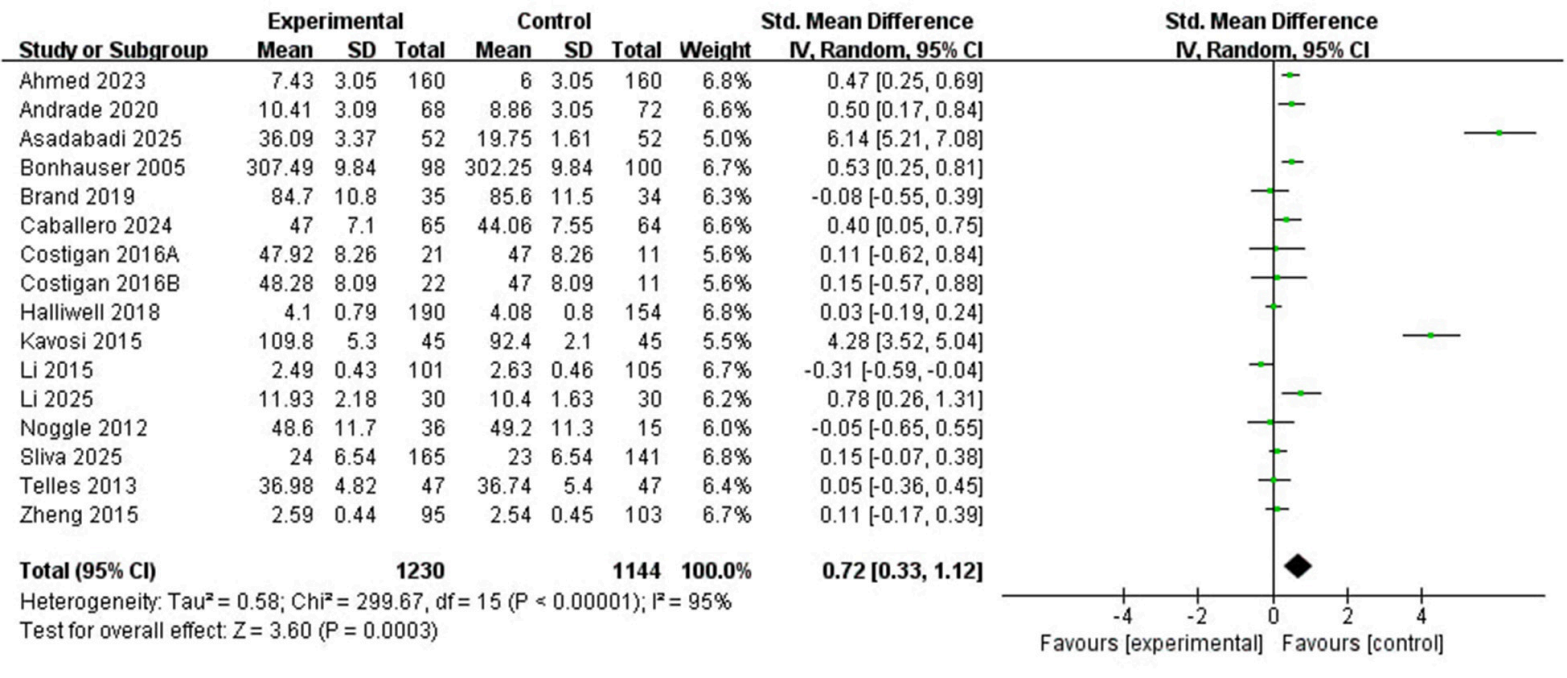
Forest plot of the relationship between positive emotions and PA in children and adolescents.

In evaluating the impact of physical activity on positive emotional and related psychological outcomes among children and adolescents, 15 studies comprising 16 comparisons were included in the meta-analysis. Because substantial heterogeneity was observed, a random-effects model was used. As shown in Figure 4, the pooled analysis indicated that physical activity interventions were associated with a statistically significant improvement in positive emotional and related psychological outcomes relative to the control conditions (SMD = 0.72, 95% CI 0.33 to 1.12, *Z* = 3.60, *P* = 0.0003). However, heterogeneity was substantial (*X*^2^ = 299.67, df = 15, *P* < 0.00001; *I*^2^ = 95%), indicating considerable between-study variability. Therefore, the pooled estimate should be interpreted cautiously, and subgroup analyses were conducted to explore possible sources of heterogeneity.

#### 3.4.2 Subgroup analysis

In order to further explore the potential changes in the impact of physical activity on positive emotions, we conducted subgroup analyses of the data (Table 3) to assess the influence of specific factors on the emotions of children and adolescents. Session length was categorized with reference to public health recommendations for youth physical activity, particularly the World Health Organization guidelines, which recommend an average of at least 60 min per day of moderate-to-vigorous physical activity for children and adolescents (WHO, 2020); the U.S. Physical Activity Guidelines for Americans were also consulted as a supplementary reference when defining the subgroup thresholds (Piercy et al., 2018). According to the recommendations of relevant guidelines, children and adolescents are required to complete at least 60 min of moderate- to high-intensity aerobic exercise every day. Based on these recommendations, subgroup analyses were conducted to further explore differences in the impact of different exercise intervention types on positive emotions (psychological wellbeing, positive mood, self-esteem, and self-efficacy), the duration of PA intervention (≥12 weeks and <12 weeks), the types of PA intervention (aerobic exercise, mind-body exercise, and mixed exercise), the control-group (NT, TAU, and AP), session length (<30 min, 30–60 min, and >60 min) and the age group (≤ 12 years and 13–25 years).

##### 3.4.2.1 Type of positive emotional outcomes

**Figure 5:**
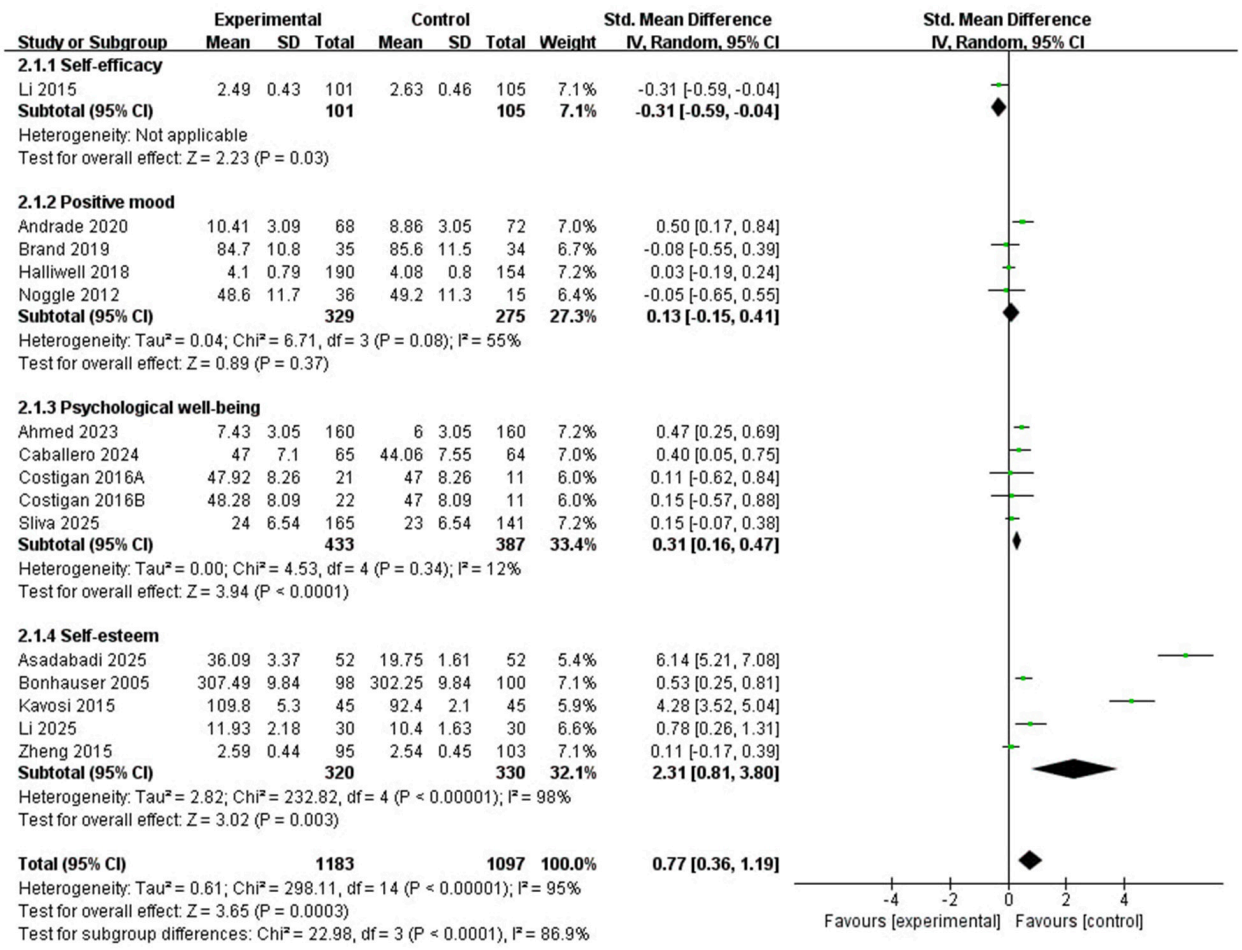
Subgroup forest plot of the relationship between PA and type of outcomes in children and adolescents.

A total of 15 studies were included (Figure 5). One study (Li et al., 2015) provided data on the effect of physical activity interventions on the self-efficacy of children and adolescents; four studies (Andrade et al., 2020; Brand et al., 2019; Halliwell et al., 2018; Noggle et al., 2012) provided data on the impact of physical activity interventions on the positive mood of children and adolescents. Four studies (Ahmed et al., 2023; Castellote-Caballero et al., 2024; Costigan et al., 2016; da Silva et al., 2025) provided data on the effect of exercise interventions on the psychological wellbeing of children and adolescents, and six studies (Asadabadi and Karami, 2025; Bonhauser et al., 2005; Kavosi et al., 2015; Li and Zhou, 2025; Telles et al., 2013; Zheng et al., 2015) provided data on the effect on self-esteem. All four aspects reflect the positive emotions of children and adolescents. Therefore, the above literature was included. The pooled results showed a statistically significant effect for self-efficacy (SMD = −0.31, 95% CI: −0.59 to −0.04, *P* = 0.03); however, because this estimate was based on a single study and the effect direction differed from most other outcome domains, it should be interpreted with particular caution and checked against the original scale coding. A significant pooled effect was also observed for psychological wellbeing (SMD = 0.31, 95% CI: 0.16 to 0.47, *P* < 0.0001; *I*^2^ = 12%) and self-esteem (SMD = 2.31, 95% CI: 0.81 to 3.80, *P* = 0.003; *I*^2^ = 98%). By contrast, the pooled effect for positive mood did not reach statistical significance (SMD = 0.13, 95% CI: −0.15 to 0.41, *P* = 0.37; *I*^2^ = 55%). The between-subgroup difference was statistically significant (*X*^2^ = 22.98, df = 3, *P* < 0.0001), suggesting that the pooled effects varied across outcome domains.

##### 3.4.2.2 Duration of physical activity intervention

**Figure 6:**
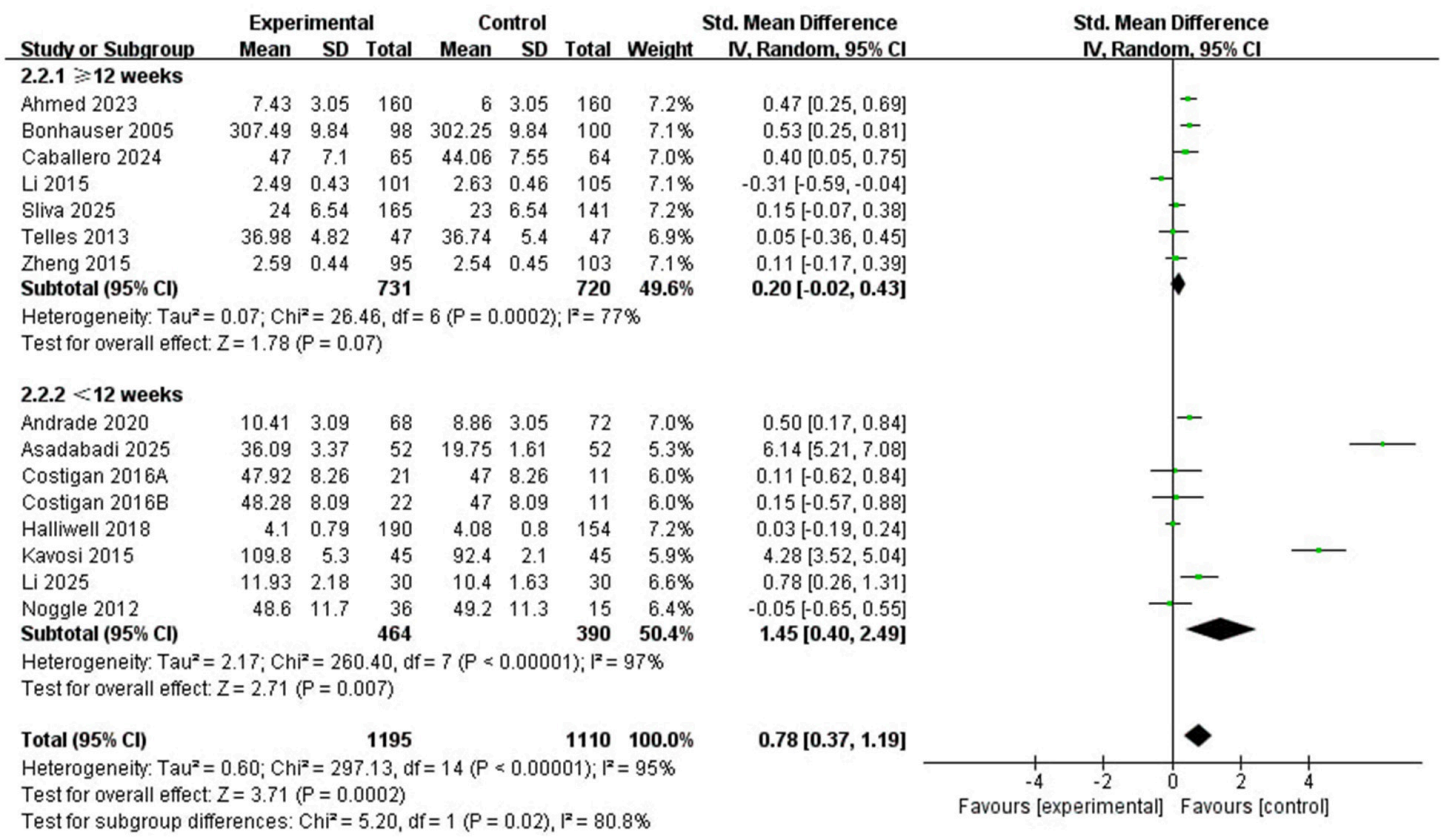
Subgroup forest plot of the relationship between PA intervention duration and positive emotions in children and adolescents.

This subgroup analysis consisted of two categories (Figure 6). 7 studies (Ahmed et al., 2023; Bonhauser et al., 2005; Castellote-Caballero et al., 2024; da Silva et al., 2025; Li et al., 2015; Telles et al., 2013; Zheng et al., 2015) focused on the intervention duration of ≥12 weeks, and 7 studies (Andrade et al., 2020; Asadabadi and Karami, 2025; Costigan et al., 2016; Halliwell et al., 2018; Kavosi et al., 2015; Li and Zhou, 2025; Noggle et al., 2012) reported interventions lasting <12 weeks. The pooled effect was statistically significant for interventions shorter than 12 weeks (SMD = 1.45, 95% CI: 0.40 to 2.49, *P* = 0.007; *I*^2^ = 97%), whereas the pooled effect for interventions lasting 12 weeks or longer did not reach statistical significance (SMD = 0.20, 95% CI: −0.02 to 0.43, *P* = 0.07; *I*^2^ = 77%). The between-subgroup difference was statistically significant (*X*^2^ = 5.20, df = 1, *P* = 0.02), suggesting that intervention duration may be associated with variation in the pooled effects. However, given the substantial heterogeneity, these findings should be interpreted cautiously.

##### 3.4.2.3 Types of physical activity intervention

**Figure 7:**
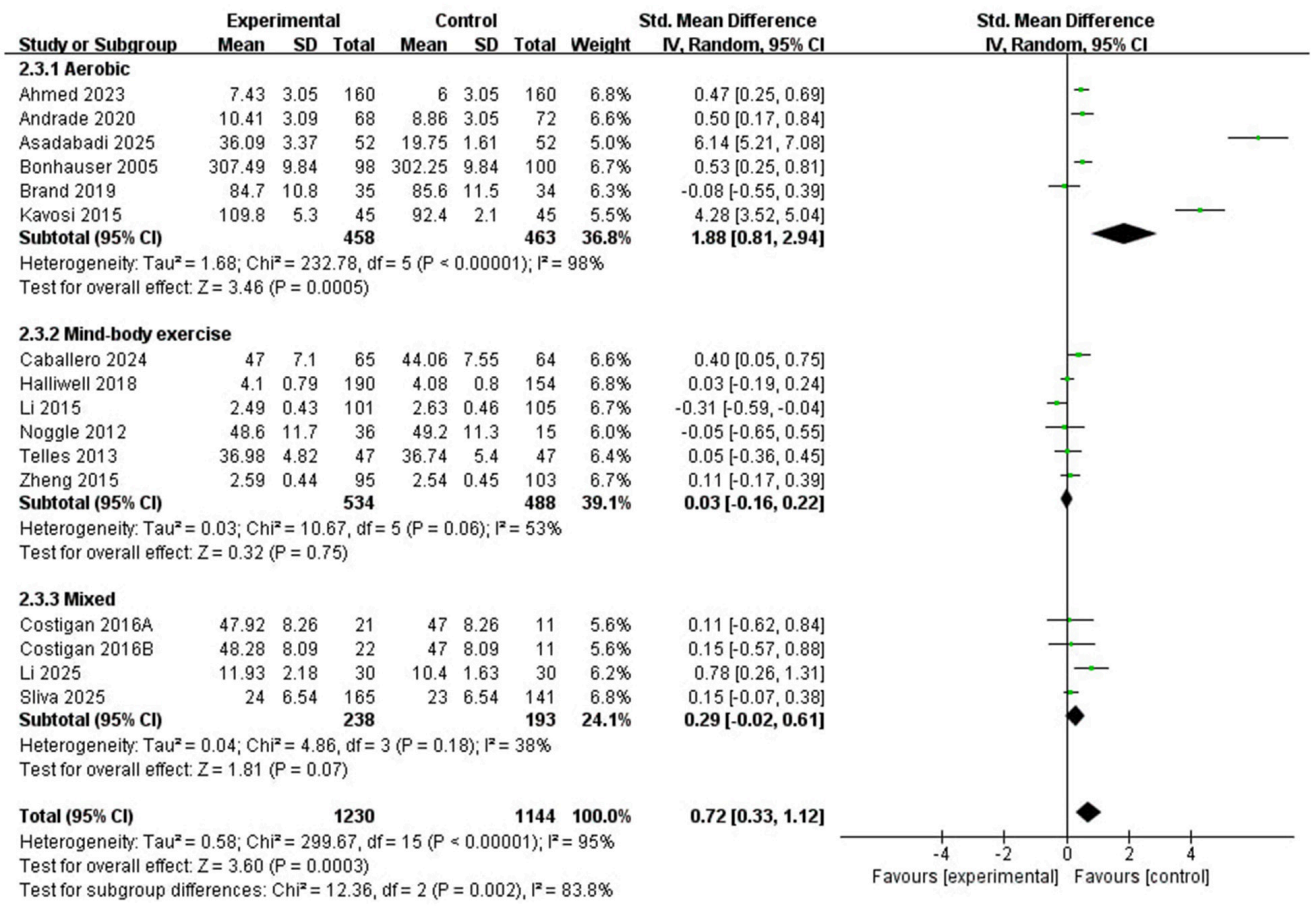
Subgroup forest plot of the relationship between types of PA intervention and positive emotions in children and adolescents.

This subgroup included a total of 15 studies (Figure 7). The 15 studies (Ahmed et al., 2023; Andrade et al., 2020; Asadabadi and Karami, 2025; Bonhauser et al., 2005; Brand et al., 2019; Castellote-Caballero et al., 2024; Costigan et al., 2016; da Silva et al., 2025; Halliwell et al., 2018; Kavosi et al., 2015; Li et al., 2015; Li and Zhou, 2025; Noggle et al., 2012; Telles et al., 2013; Zheng et al., 2015) provided data on the impact of different intervention types on positive emotions in children and adolescents, including aerobic, mind-body, and mixed types. Interventions such as yoga, Tai Chi, and Baduanjin were grouped as mind-body exercise because they share several core features, including coordinated bodily movement, breathing regulation, and attentional or meditative components. However, these interventions still differ in content, intensity, and delivery, and the corresponding subgroup findings should therefore be interpreted with caution. This grouping was adopted to facilitate synthesis, but it inevitably reduces important intervention-specific differences. Therefore, the pooled estimate for the mind-body category should be interpreted as a broad summary rather than as evidence for any single modality. The pooled effect was statistically significant for aerobic exercise (Ahmed et al., 2023; Andrade et al., 2020; Asadabadi and Karami, 2025; Bonhauser et al., 2005; Brand et al., 2019; Kavosi et al., 2015) (SMD = 1.88, 95% CI: 0.81 to 2.94, *P* = 0.0005; I^2^ = 98%). By contrast, neither mind-body exercise (Castellote-Caballero et al., 2024; Halliwell et al., 2018; Li et al., 2015; Noggle et al., 2012; Telles et al., 2013; Zheng et al., 2015) (SMD = 0.03, 95% CI: −0.16 to 0.22, *P* = 0.75; *I*^2^ = 53%) nor mixed exercise (Costigan et al., 2016; da Silva et al., 2025; Li and Zhou, 2025) (SMD = 0.29, 95% CI: −0.02 to 0.61, *P* = 0.07; *I*^2^ = 38%) showed a statistically significant pooled effect. The between-subgroup difference was statistically significant (*X*^2^ = 12.36, df = 2, *P* = 0.002), suggesting that pooled effects varied across intervention modalities. However, these subgroup findings should not be interpreted as evidence that one modality is definitively superior, because the categories were examined separately and substantial heterogeneity remained, particularly in the aerobic subgroup.

##### 3.4.2.4 Types of control groups

**Figure 8:**
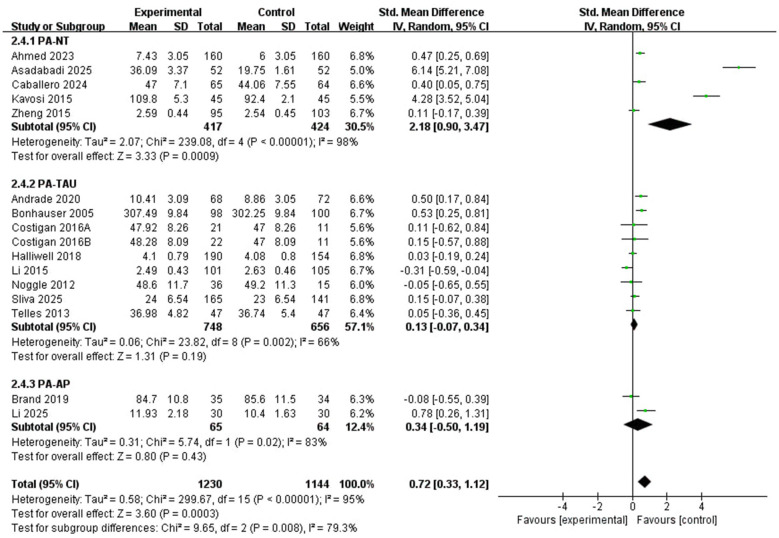
Subgroup forest plot of the relationship between PA intervention and types of control groups in children and adolescents.

A total of 15 studies were included in the analysis (Figure 8). Among these, 5 studies (Ahmed et al., 2023; Asadabadi and Karami, 2025; Castellote-Caballero et al., 2024; Kavosi et al., 2015; Zheng et al., 2015) contributed data on the nature of the no-treatment (NT) control group, 8 studies (Andrade et al., 2020; Bonhauser et al., 2005; Costigan et al., 2016; da Silva et al., 2025; Halliwell et al., 2018; Li et al., 2015; Noggle et al., 2012; Telles et al., 2013) reported data concerning the treatment-as-usual (TAU) control condition, and the remaining 2 studies (Brand et al., 2019; Li and Zhou, 2025) furnished data regarding the active placebo (AP) control group. A statistically significant pooled effect was observed in studies using no-treatment controls (SMD = 2.18, 95% CI: 0.90 to 3.47, *P* = 0.0009; *I*^2^ = 98%). In contrast, the pooled effects did not reach statistical significance in studies using treatment-as-usual controls (SMD = 0.13, 95% CI: −0.07 to 0.34, *P* = 0.19; *I*^2^ = 66%) or attention/activity placebo controls (SMD = 0.34, 95% CI: −0.50 to 1.19, P = 0.43; *I*^2^ = 83%). The between-subgroup difference was statistically significant (*X*^2^ = 9.65, df = 2, *P* = 0.008), suggesting that the apparent intervention effect varied according to the comparison condition.

##### 3.4.2.5 Time of physical activity intervention

**Figure 9:**
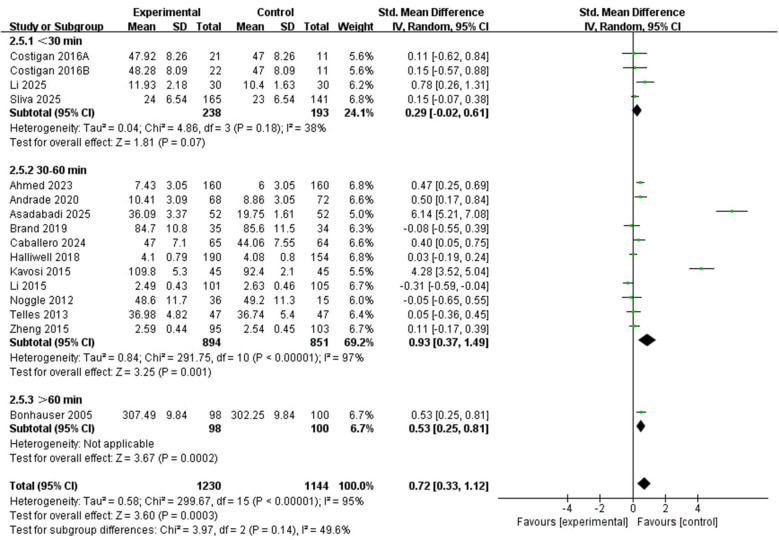
Subgroup forest plot of the relationship between time of PA intervention and positive emotions in children and adolescents.

This subgroup included a total of 15 studies (Figure 9). Three studies (Costigan et al., 2016; da Silva et al., 2025; Li and Zhou, 2025) provided data on intervention times less than 30 min; 11 studies (Ahmed et al., 2023; Andrade et al., 2020; Asadabadi and Karami, 2025; Brand et al., 2019; Castellote-Caballero et al., 2024; Halliwell et al., 2018; Kavosi et al., 2015; Li et al., 2015; Noggle et al., 2012; Telles et al., 2013; Zheng et al., 2015) provided data on intervention times between 30 and 60 min, and one (Bonhauser et al., 2005) provided data on intervention times greater than 60 min. The results indicated that the pooled effect was statistically significant for sessions lasting 30–60 min (SMD = 0.93, 95% CI: 0.37 to 1.49, *P* = 0.001; *I*^2^ = 97%). The pooled effect for sessions shorter than 30 min did not reach statistical significance (SMD = 0.29, 95% CI: −0.02 to 0.61, *P* = 0.07; *I*^2^ = 38%). A statistically significant effect was also observed for sessions longer than 60 min (SMD = 0.53, 95% CI: 0.25 to 0.81, *P* = 0.0002), but this subgroup was based on a single study and therefore should be interpreted very cautiously. The between-subgroup difference was not statistically significant (*X*^2^ = 3.97, df = 2, *P* = 0.14), indicating that session length did not significantly moderate the effect in this analysis.

##### 3.4.2.6 Age groups

**Figure 10:**
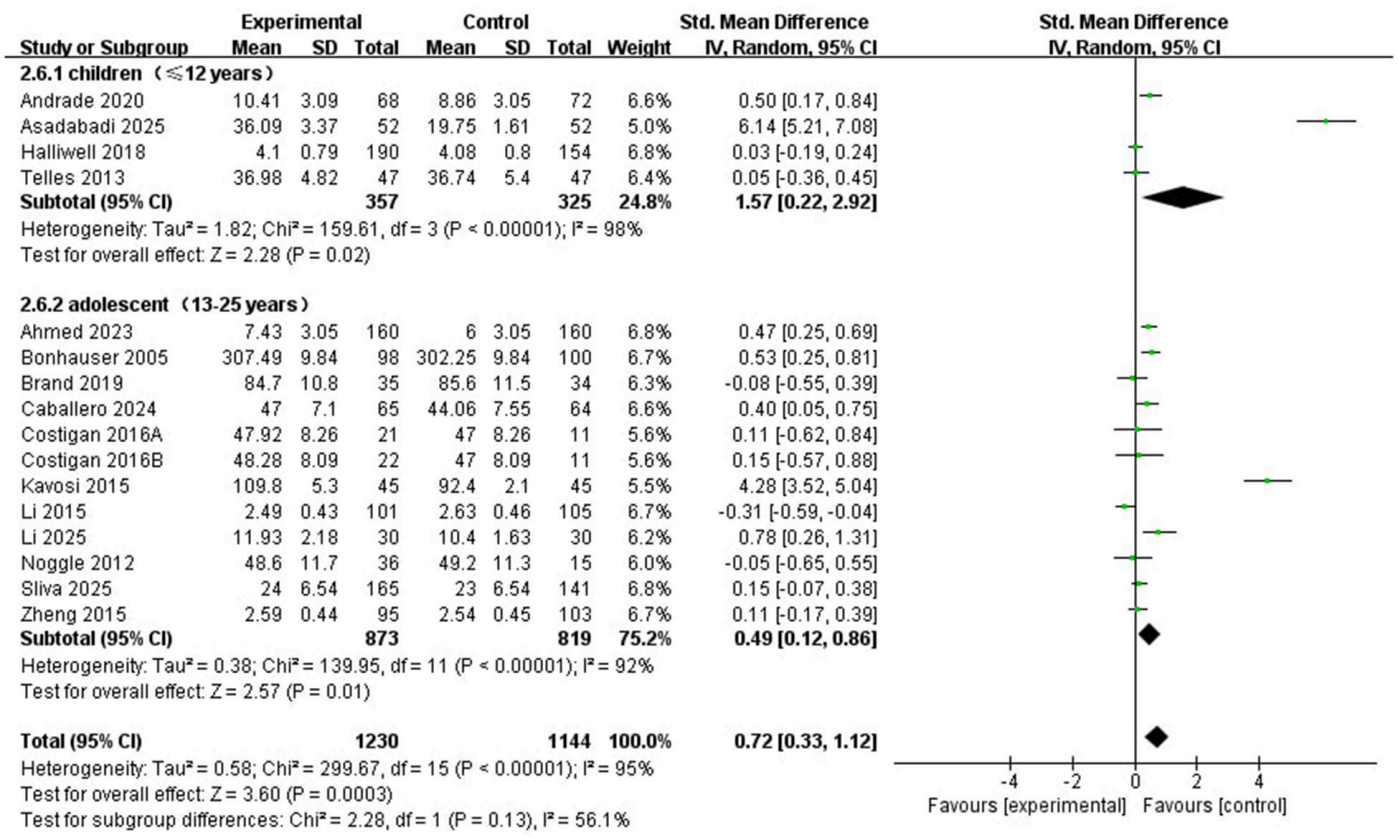
Subgroup forest plot of the relationship between time of PA intervention and types of age group in children and adolescents.

In the age-based subgroup analysis (Figure 10), statistically significant pooled effects were observed in both children aged ≤ 12 years (SMD = 1.57, 95% CI: 0.22 to 2.92, *P* = 0.02; *I*^2^ = 98%) and participants aged 13–25 years (SMD = 0.49, 95% CI: 0.12 to 0.86, *P* = 0.01; *I*^2^ = 92%). Although the pooled estimate was numerically larger in the younger subgroup, the between-subgroup difference was not statistically significant (*X*^2^ = 2.28, df = 1, *P* = 0.13), suggesting that age group did not significantly moderate the pooled effect in this analysis.

### 3.5 Sensitivity analysis

A sensitivity analysis was conducted to further explore the sources of heterogeneity. This process involves systematically excluding individual studies from the analysis to assess the impact of each study on the overall results. Leave-one-out sensitivity analyses showed that exclusion of any single study did not materially alter the direction or statistical significance of the pooled effect, suggesting that the main findings were not driven by any single trial. However, heterogeneity remained high after sequential exclusion, indicating that between-study variability likely reflected the combined influence of multiple study characteristics.

## 4 Discussion

Because intervention duration, session length, exercise type, and age were examined in separate subgroup analyses rather than in a joint or multivariable model, these findings should not be interpreted as identifying a single optimal combination of intervention characteristics. This review provided a comprehensive quantitative synthesis of several individual studies examining physical activity and positive emotional outcomes in children and adolescents through a meta-analysis, aiming to explore the impact of exercise interventions on the positive emotions of children and adolescents. We used meta-analysis to reduce the influence of bias and random error from individual studies, thereby improving the overall robustness of the findings (Zlowodzki et al., 2007). The pooled results suggested that physical activity interventions were associated with improvements in positive emotional and related psychological outcomes among children and adolescents (SMD = 0.72, 95% CI: 0.33 to 1.12, *P* = 0.0003), although substantial heterogeneity remained (*I*^2^ = 95%). The results of this review are similar to those of McMahon et al., who conducted a study on the association between sports and the wellbeing of European adolescents (McMahon et al., 2017), and are consistent with the existing research conclusions. This further supports the role of exercise in promoting mental health. These findings may be interpreted in light of broaden-and-build theory, social cognitive theory, and the conceptual model proposed by Lubans et al., which together suggest that physical activity may enhance positive outcomes through neurobiological stimulation, mastery experiences, and social connectedness rather than through a single pathway (Fredrickson, 2004; Lubans et al., 2016). Subgroup analyses indicated that statistically significant pooled effects were observed for aerobic exercise and for interventions lasting less than 12 weeks. A significant pooled effect was also found in the 30–60 min session subgroup, although the between-subgroup difference for session length was not statistically significant. Leave-one-out sensitivity analyses showed that exclusion of any single study did not materially alter the overall direction or statistical significance of the pooled estimate. These findings should therefore be interpreted cautiously in light of the heterogeneity and the separate nature of the subgroup analyses.

### 4.1 The influence of physical activity on different positive emotions of children and adolescents

Meta-analysis results suggested that the pooled effects of physical activity differed across outcome domains. Statistically significant pooled effects were observed for psychological wellbeing and self-esteem, whereas the direct effect on positive mood did not reach statistical significance, which may be related to the heterogeneity of measurement tools and concept definitions. A statistically significant result was also observed for self-efficacy; however, because this estimate was derived from a single study and showed a negative direction, it should be interpreted with particular caution. Self-efficacy, as an individual's belief in their ability to control their own behavior, may theoretically be influenced by physical exercise through mechanisms such as skill acquisition, goal achievement, and perception of physical ability, which is supported by social cognitive theory. This is also consistent with the research of Cataldo et al. (2013), who suggested that physical activity interventions may improve the self-efficacy of children and adolescents. The significant improvement in psychological wellbeing in sports activities may be related to the promotion of cognitive function, stress relief, and social connection by physical exercise (Biddle et al., 2019). The heterogeneity for psychological wellbeing in this analysis was relatively low (*I*^2^ = 12%). A significant pooled effect was also observed for self-esteem, although heterogeneity remained substantial (*I*^2^ = 98%). Future research needs to further clarify the mechanism of action and influencing factors, as well as explore the impact of factors such as age, gender, developmental status, social and cultural orientation, and external intervention on the development of self-esteem in children and adolescents (Anderson and Olnhausen, 1999). It is particularly worth noting that physical exercise did not show a significant effect on directly measured positive mood in children and adolescents. This may partly reflect variation in the definition and measurement of positive mood across studies, including the use of different instruments such as PANAS and BRUMS. In addition, as a highly fluctuating emotional state, mood is more susceptible to short-term situational factors and may be less stable than relatively stable psychological constructs (Pressman et al., 2019), such as self-efficacy or self-esteem. Overall, these findings suggest that physical activity may not affect all positive emotions outcomes to the same extent, and differences in outcome constructs and measurement approaches should be considered when interpreting the results.

### 4.2 The influence of the duration of physical activity intervention on positive emotions of children and adolescents

This review employed subgroup analysis to investigate the effects of physical exercise on positive emotional and related psychological outcomes in children and adolescents across varying intervention durations. The pooled results suggested a statistically significant effect for interventions lasting less than 12 weeks, whereas the pooled effect for interventions lasting 12 weeks or longer did not reach statistical significance. A significant between-subgroup difference was observed, suggesting that intervention duration may be associated with variation in the pooled effects. This pattern is broadly consistent with previous reviews suggesting that shorter exercise programs may still be associated with beneficial emotional outcomes (Biddle and Asare, 2011). It is also consistent with prior work noting that shorter programs may be more feasible in school- and community-based settings (Rodriguez-Ayllon et al., 2019). However, these findings should be interpreted cautiously because substantial heterogeneity remained in both duration subgroups, indicating that factors beyond duration—such as exercise type, intensity, frequency, baseline psychological status, and sociocultural context—may also contribute to variation in intervention effects. As emphasized by Ahn and Fedewa (2011) in a meta-analysis, the psychological benefits of exercise interventions are shaped by a combination of contextual and individual factors. The high heterogeneity among the included studies may stem from variations in measurement tools, sample characteristics, intervention protocols, and comparator conditions.

### 4.3 The influence of the physical activity types on positive emotions of children and adolescents

Subgroup analysis by intervention type suggested that pooled effects may vary across exercise modalities. A statistically significant pooled effect was observed for aerobic exercise, whereas mixed exercise and mind-body exercise did not reach statistical significance. The non-significant pooled effect for mind-body exercise should not be interpreted as evidence of no benefit. Instead, they may reflect heterogeneity within these categories, as interventions grouped under the same label may differ in movement patterns, intensity, delivery format, participant engagement, and cultural context. For example, yoga, Tai Chi, and Baduanjin share mind-body characteristics but differ in important practical features. Likewise, mixed exercise interventions may vary substantially in content and implementation, which could influence the observed pooled effects. These interventions were grouped together pragmatically as mind-body exercise for subgroup analysis, although they are not identical in content or delivery. The pooled effect observed for aerobic exercise is broadly consistent with previous research suggesting that aerobic activity may support emotional outcomes through physiological and psychosocial pathways (Biddle and Asare, 2011). Previous research has also suggested that more comprehensive physical activity programs may be associated with broader mental health benefits in adolescents. By contrast, mind-body exercise did not show a statistically significant pooled effect in this analysis (Singh et al., 2023). This may reflect not only the emphasis of these activities on internal awareness and relaxation, but also differences in implementation quality, participant engagement, and the time required for psychological benefits to emerge. Differences in emotional outcome measures may also have influenced the observed pooled effects (Khoury et al., 2013). Overall, variation across intervention types may reflect the combined influence of exercise modality, participant characteristics, intervention duration, and measurement approaches rather than the clear superiority of any single type.

### 4.4 The influence of the control-group types on positive emotions of children and adolescents

In this review, control-group type may have been associated with variation in the pooled effects of physical activity interventions on positive emotional and related psychological outcomes in children and adolescents. Subgroup analysis suggested that a statistically significant pooled effect was observed in studies using no-treatment (NT) controls, whereas the pooled effects did not reach statistical significance in studies using treatment-as-usual (TAU) or active placebo (AP) controls. This pattern may partly reflect differences in the nature of the comparison conditions. In particular, TAU conditions may already include some level of routine activity, psychological support, or behavioral management, thereby reducing between-group differences. Likewise, AP conditions may control for attention, social contact, or structured engagement, which could also attenuate the apparent added effect of physical activity. Previous studies have suggested that physical activity may influence emotional outcomes through multiple pathways, including neurobiological responses, enhanced self-efficacy, and increased opportunities for social interaction (Biddle and Asare, 2011; Lubans et al., 2016). In the TAU subgroup, physical exercise did not show a significant effect, which may indicate that the apparent added value of physical activity differs depending on whether participants are already receiving other forms of psychological or behavioral support (Carter et al., 2021). Some overlap in mechanisms may also exist between physical activity and psychological interventions, particularly in domains such as attention regulation and self-related cognition (Schuch et al., 2016). Overall, these findings suggest that comparator selection should be taken into account when interpreting the pooled effects of physical activity interventions.

### 4.5 The influence of session length on positive emotional outcomes in children and adolescents

The subgroup analysis of session length suggested some variation in pooled effects across categories, with a statistically significant pooled effect observed for sessions lasting 30–60 min. By contrast, the pooled effect for sessions shorter than 30 min did not reach statistical significance in the present analysis. The 30–60 min subgroup included the largest number of studies and showed a significant pooled effect. This pattern is broadly consistent with public health recommendations and previous empirical research. For example, public health guidelines and related reviews have suggested that approximately 60 min of daily moderate-to-vigorous physical activity is beneficial for the physical and mental health of children and adolescents (Poitras et al., 2016). Although the subgroup with sessions shorter than 30 min did not show a statistically significant pooled effect in this analysis, earlier work has suggested that relatively brief exercise bouts may still benefit mood-related outcomes in some contexts (Basso and Suzuki, 2017). By contrast, the subgroup with sessions longer than 60 min included only one study, so no stable inference can be drawn despite the significant result. Strong's early evidence review also suggested that for teenagers, there is no linear growth relationship between prolonged continuous exercise time and psychological benefits (Strong et al., 2005). Overall, the findings suggest that sessions of 30–60 min may be associated with positive emotional and related psychological outcomes, whereas the evidence for shorter and longer sessions remains more limited. However, these subgroup results should not be interpreted as establishing a single optimal session length, especially given the uneven number of studies across categories and the non-significant between-subgroup difference.

### 4.6 The influence of physical activity on the positive emotions of children and adolescents of different age group

This finding should be interpreted with caution. It may suggest that physical activity interventions can be associated with positive emotional and related psychological outcomes across a broad developmental period from childhood to early adulthood. This is broadly consistent with Lubans' theoretical review, which proposed that physical activity may influence emotions through multiple shared pathways, including perceived control, social connection, attentional diversion, and neurobiological responses (Lubans et al., 2016). However, heterogeneity remained very high in both age subgroups (*I*^2^ = 98% in the younger subgroup and *I*^2^ = 92% in the older subgroup), indicating that a simple dichotomization into “ ≤ 12 years” and “13–25 years” may be insufficient to capture age-related variation in emotional responses to physical activity. Therefore, the absence of a significant between-group difference does not mean that exercise experiences or intervention needs are the same across age groups. For younger children, emotional responses to physical activity may be more closely tied to playfulness, enjoyment, and opportunities for basic motor skill development. Previous reviews have similarly suggested that, for children, less structured and more game-like activities may be particularly relevant to mental health outcomes (Eime et al., 2013). For older participants, physical activity may be more closely connected to stress management, body image, peer relationships, and broader identity-related concerns. This is also consistent with prior work emphasizing that physical activity interventions for adolescents should consider autonomy, social needs, and body-related concerns (Biddle et al., 2019). In summary, although this meta-analysis did not identify a statistically significant difference in pooled effects between the age subgroups, developmental-stage considerations may still be relevant when designing physical activity interventions.

## 5 Limitations and prospects

Several limitations should be acknowledged. First, substantial heterogeneity remained in the overall analysis and in several subgroup analyses, indicating considerable between-study variability. Second, some subgroup estimates were based on a limited number of studies, particularly the >60-min subgroup and the self-efficacy subgroup, each of which included only one study. Third, although study selection was conducted independently by two reviewers and disagreements were resolved through discussion with a third reviewer, proportion agreement was not formally recorded during the screening process. This should be acknowledged as a methodological limitation. At the same time, to maximize the sensitivity and comprehensiveness of the search and to reduce the risk of omitting potentially relevant studies, the review adopted a broad retrieval and screening strategy. Although this approach strengthened the inclusiveness of the review, it limited our ability to provide a quantitative indicator of reviewer agreement. In addition, potential publication bias and incomplete reporting of important moderators, such as exercise intensity, delivery context, and individual characteristics, may also have influenced the findings.

Future research should prioritize high-quality randomized controlled trials with clearer intervention reporting and more standardized assessment of positive psychological outcomes. Greater attention should also be paid to comparator conditions, developmental-stage differences, and the potential mechanisms through which different forms of physical activity may influence psychological outcomes.

## 6 Conclusion

Physical activity interventions may improve positive emotional and related psychological outcomes in children and adolescents. Although the overall pooled effect was significant, substantial heterogeneity remained, and the subgroup findings should be interpreted cautiously. The available evidence suggests that physical activity may be a useful supportive approach for promoting positive psychological outcomes in young people, but it does not support the recommendation of a single optimal intervention pattern. Further rigorously designed studies are needed to clarify the roles of intervention type, duration, comparator condition, and developmental stage in shaping these effects.

## Data Availability

The original contributions presented in the research are included in the article/supplementary materials. For further inquiries, please contact the corresponding author directly.
